# Risk stratification and survival time of patients with gram-negative bacillary pneumonia in the intensive care unit

**DOI:** 10.3389/fcimb.2024.1382755

**Published:** 2024-05-21

**Authors:** Qiu-Xia Liao, Zhi Feng, Hui-Chang Zhuo, Ye Zhou, Peng Huang, Hai-Rong Lin

**Affiliations:** ^1^ Department of Intensive Care Unit, First Affiliated Hospital of Fujian Medical University, Fuzhou, Fujian, China; ^2^ Department of Intensive Care Unit, National Regional Medical Center, Binhai Campus of the First Affiliated Hospital, Fuzhou, Fujian, China; ^3^ Department of Thoracic Surgery, First Affiliated Hospital of Fujian Medical University, Fuzhou, Fujian, China

**Keywords:** acute kidney injury, gram-negative bacillary pneumonia, lactic acid, logistic models, nomogram

## Abstract

**Introduction:**

Pneumonia is a common infection in the intensive care unit (ICU), and gram-negative bacilli are the most common bacterial cause. The purpose of the study was to investigate the risk factors for 30-day mortality in patients with gram-negative bacillary pneumonia in the ICU, construct a predictive model, and stratify patients based on risk to assess their short-term survival.

**Methods:**

Patients admitted to the ICU with gram-negative bacillary pneumonia at Fujian Medical University Affiliated First Hospital between January 2018 and September 2020 were selected. Patients were divided into deceased and survivor groups based on whether death occurred within 30 days. Multifactorial logistic regression analysis was used to identify independent risk factors for 30-day mortality in these patients, and a predictive nomogram model was constructed based on these factors. Patients were categorized into low-, medium-, and high-risk groups according to the model's predicted probability, and Kaplan-Meier survival curves were plotted to assess short-term survival.

**Results:**

The study included 305 patients. Lactic acid (odds ratio [OR], 1.524, 95% CI: 1.057-2.197), tracheal intubation (OR: 4.202, 95% CI: 1.092-16.169), and acute kidney injury (OR:4.776, 95% CI: 1.632-13.978) were identified as independent risk factors for 30-day mortality. A nomogram prediction model was established based on these three factors. Internal validation of the model showed a Hosmer-Lemeshow test result of X2=5.770, P=0.834, and an area under the ROC curve of 0.791 (95% CI: 0.688-0.893). Bootstrap resampling of the original data 1000 times yielded a C-index of 0.791, and a decision curve analysis indicated a high net benefit when the threshold probability was between 15%-90%. The survival time for low-, medium-, and high-risk patients was 30 (30, 30), 30 (16.5, 30), and 17 (11, 27) days, respectively, which were significantly different.

**Conclusion:**

Lactic acid, tracheal intubation, and acute kidney injury were independent risk factors for 30-day mortality in patients in the ICU with gram-negative bacillary pneumonia. The predictive model constructed based on these factors showed good predictive performance and helped assess short-term survival, facilitating early intervention and treatment.

## 1 Introduction

Pneumonia is the most common infection in the intensive care unit (ICU) (Pugh et al., 2015). Currently, gram-negative bacilli are the most common pathogenic bacteria causing ICU pneumonia (Feng et al., 2023). These bacteria have a range of resistance mechanisms against antimicrobial drugs, such as increased efflux pumps, alteration of drug binding sites, reduced permeability of the bacterial outer membrane, and production of degrading enzymes, resulting in strong antibiotic resistance (Ma et al., 2009). In recent years, the incidence of pneumonia caused by gram-negative bacilli has increased, mainly due to *K. pneumoniae*, *A. baumannii*, and *P. aeruginosa* (Guzek et al., 2017; Durdu et al., 2018; Solmaz and Kalın, 2021; Assefa, 2022).

Gram-negative bacilli can be isolated from approximately 67.1% of ICU patients with pneumonia, of which *Escherichia coli* and *Pseudomonas aeruginosa* account for over 80% of these cases (Sader et al., 2018). With the widespread use of broad-spectrum antibiotics, the incidence of infections with multidrug-resistant gram-negative bacilli has increased (Cillóniz et al., 2019), significantly worsening the survival rate of infected patients. Gram-negative bacilli infections have become a major challenge to global public health (Kanj et al., 2022; Dettori et al., 2023). Studies have shown that the mortality rate of gram-negative bacillary pneumonia is 14.5%-32% (Feng et al., 2019; Kofteridis et al., 2020). Patients in the ICU are often critically ill, with shorter survival times and higher mortality rates. In addition, previous studies (Casale et al., 2023; Cheema et al., 2023) showed that death in ICU patients infected with gram-negative bacillary pneumonia is related to clinical characteristics and therapeutic measures, but the predictive efficiency of various factors varies greatly, and no effective prediction model for the short-term mortality of these patients exists, nor does an evaluation of their short-term survival time. Thus, there is an important need for predictive models to determine the short-term mortality and survival analysis of ICU patients with gram-negative bacillary pneumonia.

To bridge this gap in literature, we designed this study to retrospectively analyze the case data of ICU patients with gram-negative bacillary pneumonia, identify the risk factors for 30-day mortality, establish a predictive model, stratify the risks, and assess patient survival time. The proposed model could allow clinicians to initiate early intervention and guide clinical diagnosis and treatment, thereby improving patient therapeutic outcomes.

## 2 Materials and methods

### 2.1 Study participants

In this retrospective study conducted at the ICU of the First Affiliated Hospital of Fujian Medical University between January 2018 and September 2020, patients diagnosed with gram-negative bacillary pneumonia were included. The study’s inclusion criteria required patients to meet the diagnostic criteria for pneumonia, as outlined by Hunton (2019), and to have gram-negative bacilli cultured from their first sputum sample collected upon ICU admission. Exclusion criteria encompassed individuals under 18 years of age or those with incomplete clinical data. Additionally, to distinguish between colonization and infection, patients were excluded if they exhibited mere colonization by gram-negative bacilli. Only patients presenting clear signs of infection, such as elevated inflammatory markers and clinical symptoms consistent with infection, were included. To ensure the reliability of sputum cultures, concurrent bacterial smear tests were conducted, and sputum samples were deemed suitable for culture if they met specific criteria, including the presence of ≤10 squamous epithelial cells and ≥25 leukocytes per field under low magnification, thereby minimizing contamination risk and ensuring the clinical relevance of cultured organisms.

The endpoint of the study was either patient discharge or death. This study complied with standard medical ethics guidelines and was approved by the Medical Ethics Committee of the First Affiliated Hospital of Fujian Medical University [2015] 084–1. Informed consent was obtained from all participants.

Moreover, it should be noted that while our study focused on gram-negative bacillary pneumonia cases from January 2018 to September 2020, it is important to note that the impact of SARS-CoV-2 infection during this timeframe was likely limited due to stringent infectious disease control measures in place until January 2023.

### 2.2 Collection of clinical data

The following clinical data were collected from the patients: sex; age; underlying diseases such as hypertension, diabetes, coronary heart disease, malignant tumors, cerebral infarction, heart failure, and chronic renal failure; Charlson comorbidity index score; use of glucocorticoids, deep vein catheterization, surgery, tracheal intubation, continuous renal replacement therapy, and mechanical ventilation during hospitalization; history of acute kidney injury, duration of mechanical ventilation, liver function abnormalities, and septic shock during hospitalization; SOFA score, APACHE II score, Glasgow score, white blood cell count, platelet count, hemoglobin, procalcitonin, C-reactive protein, blood lactate, oxygenation index, creatinine, activated prothrombin time, prothrombin time, fibrinogen, and D-dimer on the first day of ICU admission.

### 2.3 Relevant definitions

The SOFA score (Arakawa et al., 2021) refers to the Sequential Organ Failure Assessment, one of the most used scores in the ICU, and is often used to assess the severity of a patient’s condition. The APACHE II score (Naved et al., 2011) consists of an acute physiology score, age score, and chronic health score and is a predictor of mortality in patients in the ICU. Acute kidney injury (Palevsky et al., 2013) referred to any of the following conditions: 1. An increase in blood creatinine ≥0.3 mg/dL (26.5 mmol/L) within 48 h; 2. An increase in blood creatinine to 1.5 times the baseline value within seven days; and 3. Urine output was <0.5 mL/kg/h, lasting for 6 h.

### 2.4 Statistical analysis

Data analysis was performed using StataSE15.0. Continuous variables that conformed to a normal distribution were expressed as mean ± standard deviation and tested using *t*-tests. Non-normally distributed continuous variables were expressed as medians (interquartile ranges) and tested using the Mann-Whitney U test.

To develop the multivariate logistic regression model, we designated the 30-day mortality of the investigated ICU patients as the dependent variable. Initially, univariate analyses were conducted to screen potential predictors of mortality, with variables showing significant associations (P < 0.05) being considered for inclusion in the multivariate model. Additionally, variables deemed clinically relevant based on professional knowledge and literature review were also considered. Then, a stepwise forward selection process with a significance level set at P < 0.05 was used to identify the final predictors included in the mode to ensure that only variables contributing significantly to the prediction of 30-day mortality were included in the final model.

Based on the obtained independent variables, a nomogram was drawn to visualize the prediction model, and a receiver operating characteristic (ROC) curve was used to evaluate the discriminative ability of the model. The Hosmer-Lemeshow test was used to determine the model’s stability, and the decision curve analysis was used to assess the clinical utility of the model. Patients were stratified by risk based on the model’s score and predicted probability and three Kaplan-Meier survival curves were drawn. Statistical significance was set at P < 0.05.

## 3 Results

### 3.1 Bacterial distribution in ICU patients with gram-negative bacillary pneumonia

The bacterial strains infecting ICU patients with gram-negative bacillary pneumonia were *Klebsiella pneumoniae* in 180 cases (59.0%), *Acinetobacter baumannii* in 73 (23.9%), *P. aeruginosa* in 28 (9.2%), *E. coli* in 14 (4.6%), *Stenotrophomonas maltophilia* in 2 (0.7%), *Serratia marcescens* in 2 (0.7%), and other gram-negative bacteria in 6 (1.9%) (**Table 1**).

**Table 1:** Bacterial Distribution in ICU Patients with Gram-Negative Bacillary Pneumonia.

Bacterial Strain	Number (N=305)	P-value
*Klebsiella pneumoniae*	180	59.0%
*Acinetobacter baumannii*	73	23.9%
*Pseudomonas aeruginosa*	28	9.2%
*Escherichia coli*	14	4.6%
*Stenotrophomonas maltophilia*	2	0.7%
*Serratia marcescens*	2	0.7%
Other gram-negative bacteria	6	1.9%

### 3.2 Baseline characteristics

**Figure 1:**
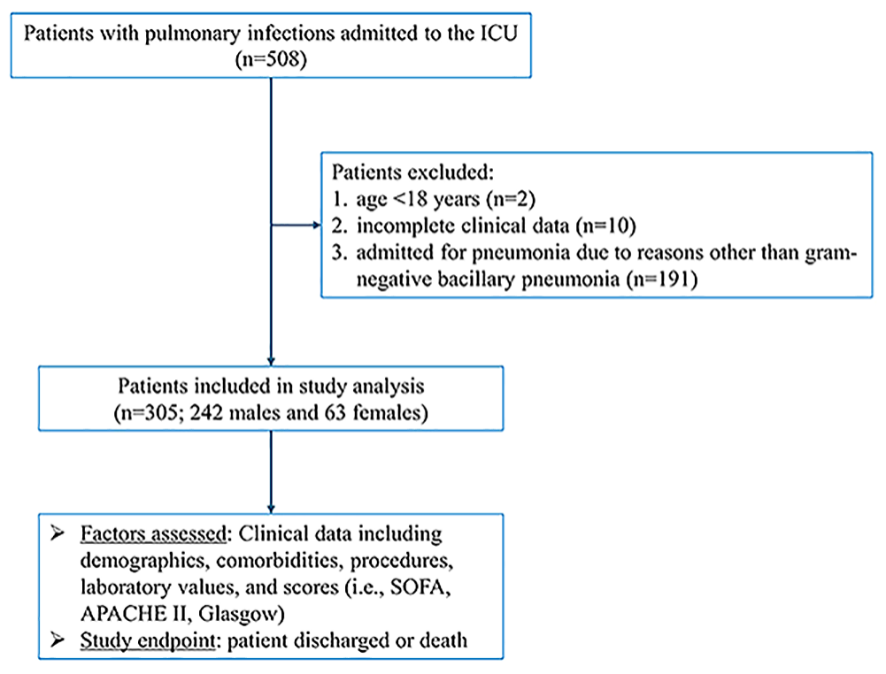
Flow chart.

During the study period from January 2018 to September 2020, a total of 508 patients with pulmonary infections were admitted to the ICU of the First Affiliated Hospital of Fujian Medical University. Among them, 2 patients were excluded due to age <18 years and 10 patients were excluded due to incomplete clinical data. Additionally, 191 patients were admitted for pneumonia due to reasons other than gram-negative bacillary pneumonia. Ultimately, 305 patients with gram-negative bacillary pneumonia were included in our study, comprising 242 males and 63 females. The study flow chart is shown in **Figure 1**.

The patients were grouped based on overall survival within 30 days, with 240 (78.7%) and 65 (21.3%) in the survivor and deceased groups, respectively. Patients in the deceased group had significantly higher rates of septic shock, hypoalbuminemia, chronic renal insufficiency, and acute kidney injury than those in the survivor group. They also had higher SOFA and APACHE II scores and an increased likelihood of undergoing invasive procedures such as central venous catheterization, tracheal intubation, and continuous renal replacement therapy. The incidence of carbapenem-resistant gram-negative bacteria was higher, hemoglobin levels were significantly lower, and procalcitonin, interleukin-6, lactate, and serum creatinine levels were markedly increased. Activated partial thromboplastin and prothrombin times were extended, D-dimer levels were elevated, mechanical ventilation time was prolonged, and hospital and ICU stays were both significantly shorter (P < 0.05). No statistically significant differences were observed between the two groups in terms of sex, age, hypertension, malignancy, coronary artery disease, diabetes, stroke, Charlson comorbidity index score, hospital-acquired pneumonia, history of corticosteroid use, surgical history, white blood cell count, C-reactive protein level, oxygenation index, bilirubin level, Glasgow Coma Scale score, or fibrinogen level (P ≥ 0.05, **Table 2**). In regard to the incidence rate of bloodstream infections, our results showed that the incidence rate of bloodstream infections was found to be 9/65 (13.85%) in the deceased group and 14/240 (5.83%) in the survivor group, indicating a significantly higher rate in the deceased group compared to the surviving group (P < 0.030).

**Table 2:** Baseline characteristics of patients in the survivor and deceased groups.

Variable	Survivor Group (N=240)	Deceased Group (N=65)	P-value
Sex (Female)	45 (18.8%)	18 (27.7%)	0.114
Age (Years)	64.00 (52.00, 75.00)	65.00 (53.00, 76.00)	0.431
Hypertension	136 (56.7%)	40 (61.5%)	0.481
Malignant Tumor	30 (12.5%)	13 (20.0%)	0.123
Coronary Heart Disease	17 (7.1%)	8 (12.3%)	0.173
Diabetes	66 (27.5%)	15 (23.1%)	0.474
Cerebral Infarction	54 (22.5%)	14 (21.5%)	0.859
Septic Shock	38 (15.8%)	21 (32.3%)	0.003
Hypoalbuminemia	89 (37.1%)	35 (53.8%)	0.015
Chronic Renal Failure	21 (8.8%)	12 (18.5%)	0.025
Heart Failure	33 (13.8%)	10 (15.4%)	0.737
Acute Kidney Injury	81 (33.8%)	51 (78.5%)	<0.001
Hospital-acquired Pneumonia	223 (92.9%)	62 (95.4%)	0.476
bloodstream infections	14(5.83%)	9(13.85%)	0.030
History of Corticosteroid Use	63 (26.3%)	19 (29.2%)	0.631
Central Venous Catheterization	119 (49.6%)	43 (66.2%)	0.018
Surgical History	114 (47.5%)	31 (47.7%)	0.978
Tracheal Intubation	161 (67.1%)	52 (80.0%)	0.044
Continuous Renal Replacement Therapy	28 (11.7%)	15 (23.1%)	0.019
Carbapenem-resistant Gram-negative Bacilli	159 (66.3%)	52 (80.0%)	0.033
Charlson Comorbidity Index score (scores)	4.00 (2.00, 6.00)	5.00 (3.00, 7.00)	0.071
SOFA Score (scores)	5.00 (4.00, 7.00)	7.00 (5.00, 11.00)	<0.001
APACHE II Score (scores)	17.00 (13.00, 21.00)	20.00 (17.00, 26.00)	<0.001
White Blood Cell Count (10^9^/L)	10.34 (7.70, 14.68)	9.41 (7.57, 14.56)	0.379
Platelet Count (10^9^/L)	194.00 (136.00, 284.00)	144.00 (89.00, 199.00)	<0.001
Hemoglobin (g/L)	103.50 (84.00, 125.00)	88.00 (72.00, 115.00)	0.004
Procalcitonin (ng/mL)	0.41 (0.13, 2.40)	3.37 (0.72, 6.80)	<0.001
C-reactive Protein (mg/L)	62.26 (29.00, 90.00)	75.84 (35.38, 90.00)	0.191
Interleukin-6 (pg/mL)	54.36 (17.57, 161.80)	263.20 (101.00, 461.10)	0.014
Lactic Acid (mmol/L)	1.68 (1.38, 2.30)	2.60 (1.70, 3.60)	0.007
Oxygenation Index	300.00 (228.00, 346.00)	285.00 (201.50, 328.00)	0.212
Serum Creatinine (µmol/L)	71.70 (50.40, 103.20)	110.60 (60.40, 211.90)	<0.001
Bilirubin (µmol/L)	11.30 (6.50, 16.40)	12.40 (7.00, 19.80)	0.295
Glasgow Coma Scale (scores)	8.00 (5.00, 10.00)	7.00 (5.00, 10.00)	0.885
Activated Partial Thromboplastin Time (seconds)	32.90 (26.40, 39.90)	38.65 (31.10, 46.25)	0.003
Prothrombin Time (seconds)	13.30 (12.10, 14.60)	14.50 (12.90, 16.55)	0.001
Fibrinogen (g/L)	3.82 (2.41, 4.99)	3.71 (2.05, 4.81)	0.379
D-Dimer (mg/L)	3.39 (1.70, 6.53)	6.60 (2.99, 13.14)	0.003
Mechanical Ventilation Duration (days)	5.00 (0.00, 13.00)	8.00 (3.00, 14.00)	0.045
Hospital Stay Duration (days)	21.00 (14.50, 29.00)	15.00 (11.00, 21.00)	<0.001
ICU Stay Duration (days)	20.00 (13.00, 27.00)	13.00 (9.00, 18.00)	<0.001

Continuous variables not following a normal distribution are represented as median (interquartile range), and categorical variables are represented as cases (%).

SOFA, Sequential Organ Failure Assessment; APACHE II, Acute Physiology and Chronic Health Evaluation; ICU, Intensive Care Unit.

### 3.3 Multifactorial logistic regression analysis

The 30-day mortality of ICU patients with gram-negative bacillary pneumonia was a dependent variable. The variables assessed included the patients’ SOFA scores, platelet counts, procalcitonin, lactic acid, and APACHE II scores on the first day of ICU admission, whether the patient developed infectious shock, hypoalbuminemia, bloodstream infections, tracheal intubation, continuous renal replacement therapy, carbapenem-resistant gram-negative Bacilli, acute kidney injury, and duration of mechanical ventilation during the hospital stay. Using stepwise forward logistic regression with a significance level of P < 0.05, we found that lactic acid (odds ratio (OR): 1.524, 95% CI: 1.057–2.197), tracheal intubation (OR: 4.202, 95% CI: 1.092–16.169), and acute kidney injury (OR: 4.776, 95% CI: 1.632–13.978) were independent risk factors for 30-day mortality in ICU patients with gram-negative bacillary pneumonia. However, upon inclusion of bloodstream infections in a multifactorial logistic regression analysis, it was revealed that bloodstream infections are not an independent risk factor for 30-day mortality in ICU patients with Gram-negative bacterial pneumonia. The equation for the model is listed below. P=1/[1+exp [-(-3.999 + 0.421X1 + 1.435X2 + 1.564X3)]] (**Table 3**).

**Table 3:** Multifactorial logistic regression analysis of 30-day mortality in ICU patients with gram-negative bacilli infections.

Variables	β	OR	95%CI	P-values
Lactic Acid (mmol/L) (X1)	0.421	1.524	1.057–2.197	0.024
Tracheal Intubation (X2)	1.435	4.202	1.092–16.169	0.037
Acute Kidney Injury (X3)	1.564	4.776	1.632–13.978	0.004

b, beta values; OR, odds ratios; CI, confidence intervals.

### 3.4 Establishment of the nomogram prediction model

**Figure 2:**
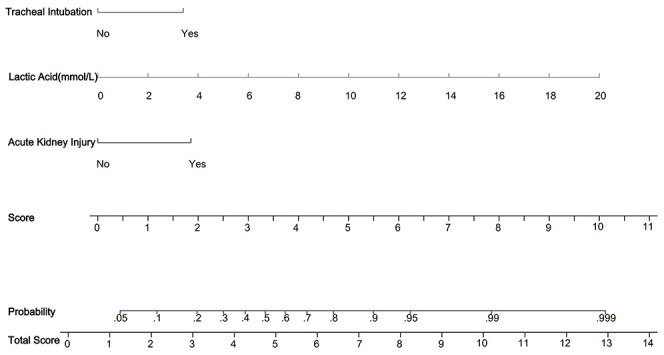
Nomogram predicting 30-day mortality in ICU patients with gram-negative pneumonia. In the figure, it can be seen that acute kidney injury (1.9 points), lactic acid (1 point for every 2 mmol increase), and tracheal intubation (1.7 points) are depicted. Then, the scores for each variable are summed to obtain the total score, which corresponds to the predicted probability of 30-day mortality in ICU patients with gram-negative bacillary pneumonia. For example, for a patient with gram-negative bacillary pneumonia in the ICU who experienced acute kidney injury, endotracheal intubation, and a lactate level of 4 mmol/L, according to the nomogram, the total score would be calculated as follows: 1.9 + 2 + 1.7 = 5.6 points, corresponding to a 66% probability of 30-day mortality.

Three independent risk factors were identified using multiple logistic regression analysis. The StataSE15.0 software was used to construct a predictive model for 30-day mortality in ICU patients with pneumonia caused by infection with gram-negative bacilli, and a nomolog graph (**Figure 2**) was created. The regression coefficients of each variable were used to calculate corresponding scores, with acute kidney injury, an increase in blood lactate by 2 mmol/L, and tracheal intubation scoring 1.9, 1, and 1.7 points, respectively. The sum of the individual scores of each variable gives a total score, which corresponds to the probability of 30-day mortality in ICU patients with gram-negative bacillary pneumonia. The total score in the model ranged from 0 to 13.6 points. For example, an ICU patient with gram-negative bacillary pneumonia who experienced acute kidney injury, tracheal intubation, and a lactate level of 4 mmol/L would have a score of 5.6 points according to the nomogram, correlating to a 66% probability of 30-day mortality.

### 3.5 Internal validation of the predictive model

**Figure 3:**
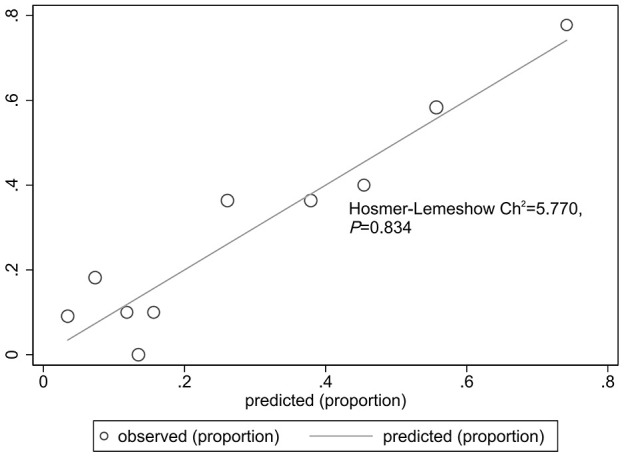
Hosmer-Lemeshow test for the predictive model.

**Figure 4:**
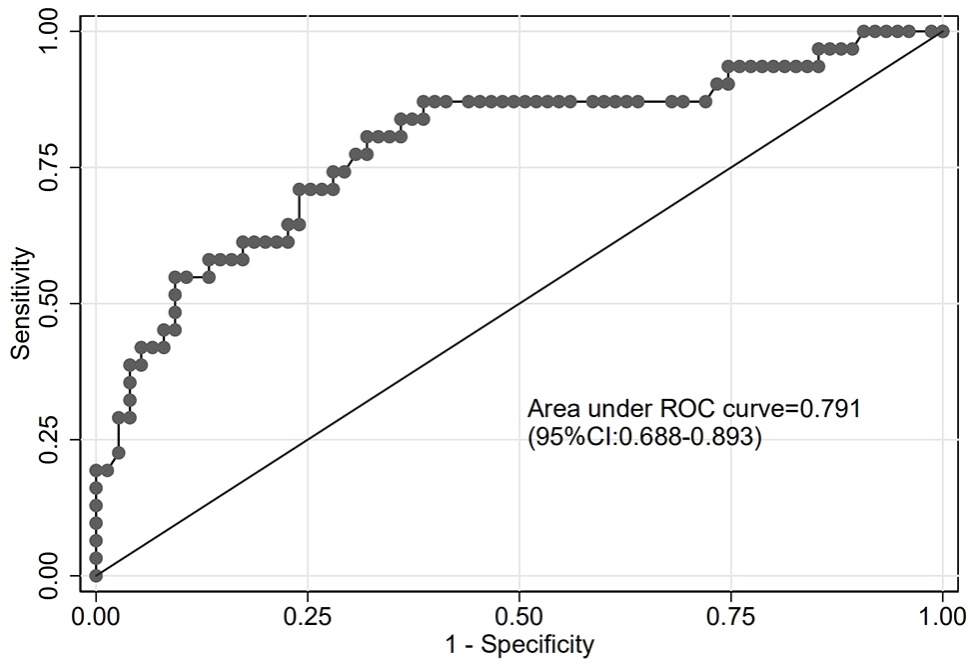
Receiver operating characteristic (ROC) curve for the predictive model.

**Figure 5:**
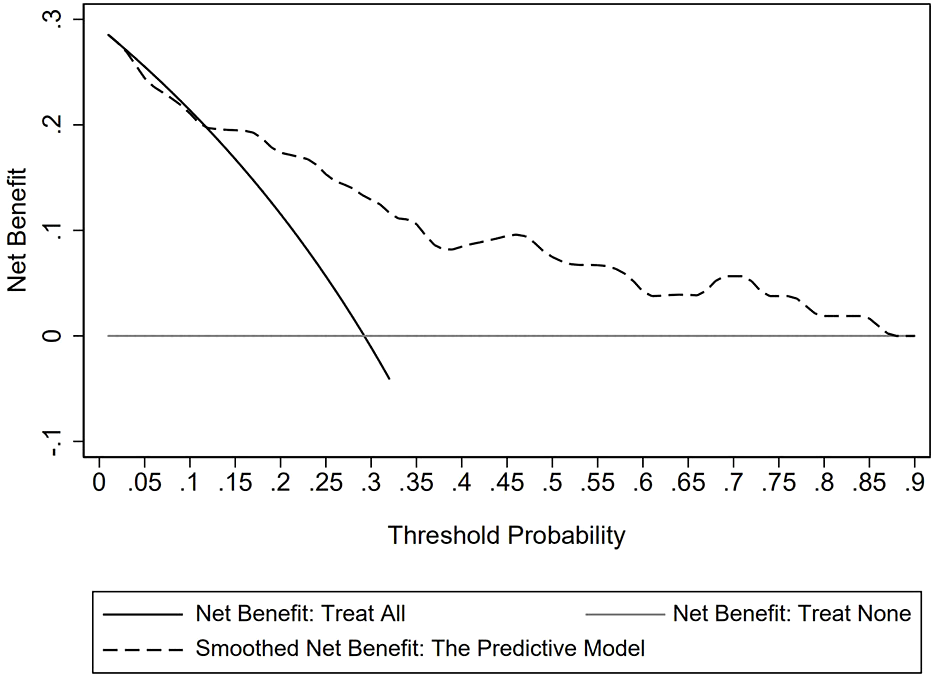
Decision curve analysis for the predictive model.

The goodness of fit of the predictive model was assessed using the Hosmer-Lemeshow test, which showed a chi-square value of 5.770 and a P-value of 0.834, indicating a good fit (**Figure 3**). After plotting the ROC curve, the area under the curve was 0.791 (95% CI: 0.688–0.893), suggesting that the predictive model was effective and had good discriminatory ability (**Figure 4**). Internal validation was performed using the bootstrap method, with 1000 repeated samples of the original data yielding a C-index of 0.791 (0.690–0.891), indicating that the model exhibited good discriminatory power. The decision curve revealed a high net benefit value for threshold probabilities between 0.15 and 0.9, indicating a high value for the predictive model (**Figure 5**).

### 3.6 Average survival time of patients with low, medium, and high risk

**Figure 6:**
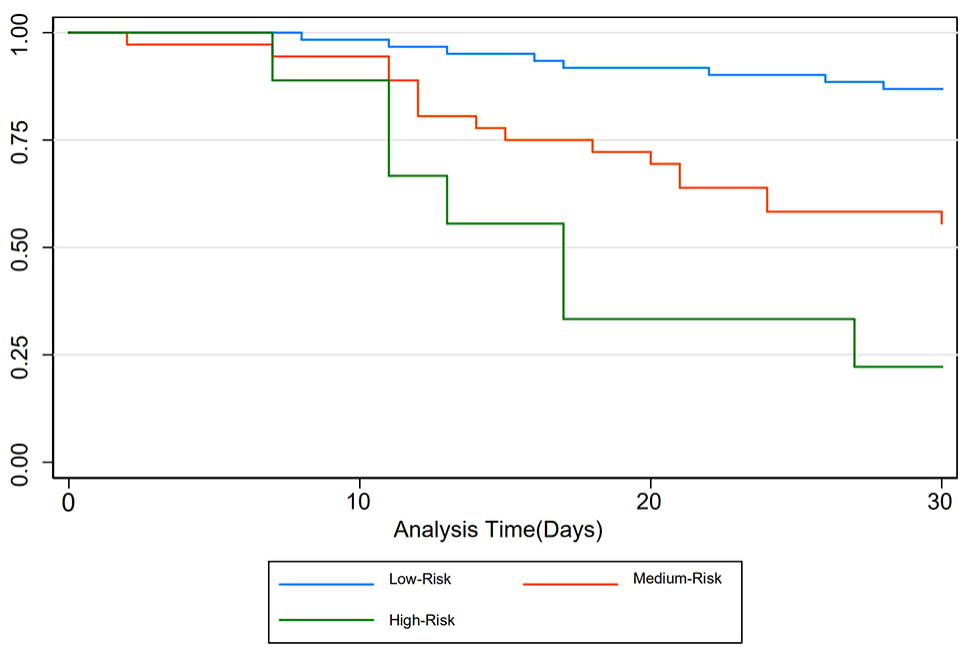
Kaplan-Meier survival curves for each risk stratification.

Patients were categorized as low-, medium-, or high-risk based on the predicted probability of death within 30 days of hospitalization: scores less than 3.8 with a predicted probability of less than 30% were considered low risk; scores between 3.8 and 5.2 with a predicted probability greater than 30% and less than 60% were considered medium risk; and scores equal to or greater than 5.2 with a predicted probability equal to or greater than 60% were considered high risk. The outcome measure was 30-day mortality in ICU patients with gram-negative pneumonia, as depicted by the Kaplan-Meier survival curve. The median survival times were 30 (30, 30) days for low-risk patients, 30 (16.5, 30) days for medium-risk patients, and 17 (11, 27) days for high-risk patients, exhibiting statistically significant differences (P < 0.001) (**Figure 6**).

## 4 Discussion

This retrospective study revealed the predominant bacterial strains causing gram-negative bacillary pneumonia in ICU patients, with Klebsiella pneumoniae being the most common pathogen identified, followed by Acinetobacter baumannii and Pseudomonas aeruginosa. Among the 305 patients included in the study, those who succumbed within 30 days exhibited significantly higher rates of septic shock, hypoalbuminemia, chronic renal insufficiency, and acute kidney injury, along with elevated SOFA and APACHE II scores. Multifactorial logistic regression analysis identified lactic acid, tracheal intubation, and acute kidney injury as independent risk factors for 30-day mortality in these patients. Moreover, a nomogram prediction model was developed, demonstrating good discriminatory ability and providing valuable insights into risk stratification for mortality prediction in ICU patients with gram-negative bacillary pneumonia. Overall, our study provides promising insights for critical care medicine, offering a robust framework for predicting mortality risk and informing targeted interventions to optimize patient care in the management of gram-negative bacillary pneumonia in the ICU.

The incidence of pneumonia caused by gram-negative bacilli has been reported to be increasing (Assefa, 2022; Solmaz and Kalın, 2021; Guzek et al., 2017; Durdu et al., 2018; Radulescu and Gant, 2023), which is consistent with the findings of this study. ICU patients are often critically ill and immunocompromised, which makes them susceptible to opportunistic infections. Once infected with gram-negative bacilli, pneumonia can develop, increasing the patient’s mortality and medical costs (Oliveira and Reygaert, 2023). Our results indicated a mortality rate of 21.3%, which aligns with a previous report showing a 23.1% mortality rate under the same conditions (Feng et al., 2019), suggesting that patients in the ICU with gram-negative bacterial pneumonia are prone to short-term mortality, and preventing and controlling the risk factors of death is essential to improving clinical outcomes.

The gender distribution observed in our study raises interesting questions regarding the epidemiology and susceptibility of gram-negative bacillary pneumonia in ICU settings in China, particularly at our institution. This gender disparity could reflect broader trends in the prevalence of risk factors for severe infections, including lifestyle factors, genetic predispositions, or access to care, which might differ significantly between genders (Mauvais-Jarvis et al., 2020; Gay et al., 2021; Migliore et al., 2021). Previous studies have suggested that males may have a higher incidence of certain risk factors for pneumonia, such as smoking and alcohol use, which could contribute to the increased susceptibility to infections, including gram-negative bacillary pneumonia (Groeneveld et al., 2020; Corica et al., 2022). Additionally, occupational exposure to pollutants or pathogens might be higher in males, depending on regional and socioeconomic contexts (Biswas et al., 2021). It’s also possible that inherent biological differences, such as immune system function, could contribute to these disparities (Biswas et al., 2021). However, our study did not explicitly explore the reasons behind this gender distribution, and further research is needed to understand the underlying causes fully.

Logistic regression is a valuable tool for assessing disease risk factors and estimating disease occurrence probabilities under varied circumstances. Our investigation identified acute kidney injury, lactic acidosis, and tracheal intubation as independent risk factors for 30-day mortality among ICU patients infected with gram-negative bacterial pneumonia. A precedent inquiry established a nexus between renal dysfunction and mortality in 96 subjects grappling with carbapenem-resistant gram-negative bacterial pneumonia (Shen et al., 2022). Given the inflammatory nature of both pneumonia and acute kidney injury, an increased immune response could manifest in pneumonia patients concomitantly experiencing acute kidney injury, thereby complicating and exacerbating the condition while drastically diminishing survival prospects (Murugan et al., 2010; Chawla et al., 2017). Previous multivariate logistic regression analysis in pneumonia patients identified lactic acid levels as an additional independent mortality risk factor (Demirel, 2018). Gram-negative bacterial pneumonia sufferers frequently encounter multi-organ failure mainly attributable to hypoxia and insufficient perfusion. Lactic acid, a byproduct of anaerobic glucose fermentation, mirrors augmented anaerobic metabolism, serving as a direct indicator of tissue hypoxia and suboptimal perfusion, intricately linked with pneumonia severity and mortality risk (Liu et al., 2015). Furthermore, investigations have demonstrated a marked escalation in mortality risk among gram-negative bacterial pneumonia patients necessitating tracheal intubation (Lodise et al., 2021), consistent with our findings. These findings imply that individuals requiring tracheal intubation often present compromised pulmonary function, severe illness and diminished resistance to infection, rendering them particularly susceptible to mortality. Thus, to enhance the outcomes of individuals requiring tracheal intubation due to gram-negative bacterial pneumonia, comprehensive supportive care, including meticulous monitoring of pulmonary function, aggressive treatment of infections, and optimization of organ perfusion, should be prioritized, aiming to reduce mortality risks associated with their compromised condition.

The nomogram chart presents a novel predictive model characterized by high accuracy and ease of dissemination. In contrast to Feng et al. (2019), who employed multivariate logistic regression analysis on 269 cases of gram-negative bacterial pneumonia to ascertain ICU admission as a mortality risk factor, our study directly focused on ICU patients afflicted with gram-negative bacterial pneumonia. By integrating clinically relevant indicators into the nomogram chart and employing multivariate logistic regression analysis to amalgamate multiple predictive indicators, complex data are transformed into a visually accessible format, enhancing the intuitive, rigorous, and practical aspects of the prediction model for patient risk assessment. Unlike studies such as Baek et al. (2020), which solely utilized the area under the ROC curve to validate mortality predictive models in elderly patients with severe pneumonia, our research employed multiple validation methods, augmenting the credibility of the model. The Hosmer-Lemeshow test results affirm the good fit of the predictive model. With an area under the ROC curve of 0.791, our predictive model demonstrates effectiveness in forecasting the 30-day mortality risk among ICU patients with gram-negative bacterial pneumonia. Additionally, the decision curve analysis underscores a high net benefit and clinical value of the predictive model, further substantiating its utility in clinical practice.

Kaplan-Meier survival curve analysis serves as a method to depict survival time on the x-axis and survival rate on the y-axis, facilitating the assessment of certain factors’ impact on survival duration. In our investigation, we employed Kaplan-Meier survival curve analysis to evaluate the 30-day survival duration of patients categorized into low, medium, and high-risk groups based on the predictive model. The findings revealed a notable correlation between higher risk scores and shorter survival times. This stands in contrast to the study by Shen et al. (2022), which solely identified mortality risk factors in patients with gram-negative bacterial pneumonia without stratifying patients by risk or examining their short-term survival. Moreover, our study supplemented the findings with graphical representations of Kaplan-Meier survival curves, enhancing their interpretability and applicability for clinicians. Overall, upon further validation, our proposed nomogram could help guide interventions and optimize patient care in ICU settings. By stratifying patients based on their risk of mortality, healthcare providers can tailor interventions to address specific needs and mitigate adverse outcomes. For instance, patients identified as high risk according to the predictive nomogram model may benefit from closer monitoring, aggressive management of complications such as acute kidney injury, and early initiation of appropriate antimicrobial therapy targeting multidrug-resistant pathogens. Moreover, risk-based interventions can help allocate limited healthcare resources effectively, ensuring that high-risk patients receive timely and targeted interventions to improve their chances of survival. By incorporating risk stratification into clinical practice, healthcare providers can enhance the precision and effectiveness of interventions, ultimately improving patient outcomes in ICU settings.

Despite our efforts to comprehensively analyze risk factors for mortality in ICU patients with gram-negative bacillary pneumonia, several limitations should be acknowledged. Firstly, our study did not specifically assess the impact of antibiotic therapy on patient outcomes, which represents an important aspect of pneumonia management that could influence mortality rates. Second, the retrospective nature of our study design may have introduced selection bias and limited our ability to establish causal relationships between identified risk factors and mortality. Third, our study was conducted at a single center without external validation, potentially limiting the generalizability of our findings to other patient populations and healthcare settings. In addition, the possibility of unmeasured confounding variables influencing our results cannot be entirely ruled out. Lastly, the potential multicollinearity between APACHE and SOFA scores as independent variables could be considered a limitation, though the VIF coefficients were below the threshold of 5 (2.94 for APACHE and 2.09 for SOFA). Further investigation into the impact of these limitations on our findings could provide additional insights in future studies.

## 5 Conclusion

In conclusion, our findings reveal the important role of acute kidney injury, lactic acidosis and the need for tracheal intubation in determining the short-term mortality risk of ICU patients with gram-negative bacillary pneumonia. In regards to our proposed predictive model, this study provides important insights for devising a tool capable of early risk stratification, guiding more personalized and effective clinical interventions to improve the outcomes of these patients.

## Data availability statement

The original contributions presented in the study are included in the article/supplementary material, further inquiries can be directed to the corresponding author/s.

## Ethics statement

The studies involving humans were approved by the Medical Ethics Committee of the First Affiliated Hospital of Fujian Medical University [2015] 084-1. The studies were conducted in accordance with the local legislation and institutional requirements. The participants provided their written informed consent to participate in this study.

## Author contributions

Q-XL: Writing – original draft, Writing – review & editing, Conceptualization, Data curation, Formal analysis, Funding acquisition, Methodology, Supervision, Validation. ZF: Conceptualization, Data curation, Methodology, Writing – original draft, Writing – review & editing. H-CZ: Conceptualization, Data curation, Methodology, Supervision, Validation, Writing – original draft, Writing – review & editing. YZ: Conceptualization, Data curation, Methodology, Writing – review & editing. PH: Data curation, Methodology, Writing – review & editing. HL: Data curation, Writing – review & editing.
